# Mass Spectrometry Chromatography-Based Metabolomics: The Effect of Long-Term Aerobic Exercise on Learning Ability and the Metabolism of Intestinal Contents in Mice with Alzheimer’s Disease

**DOI:** 10.3390/metabo13111150

**Published:** 2023-11-14

**Authors:** Xue Li, Yuanting Zhang, Xianyi Ding, Yu Jin, Changling Wei, Jisheng Xu

**Affiliations:** School of Sports Medicine and Health, Chengdu Sport University, Chengdu 610041, China; yuantingz@cdsu.edu.cn (Y.Z.); 2311516008@sus.edu.cn (X.D.); jinyu@cdsu.edu.cn (Y.J.); 27053337@alu.cqu.edu.cn (C.W.); xujisheng@cdsu.edu.cn (J.X.)

**Keywords:** exercise, Alzheimer’s disease, intestinal contents, metabolome

## Abstract

This study aimed to investigate the effect of long-term aerobic exercise on the metabolism of intestinal contents in APP/PS1 mice was studied using a non-targeted metabolomics technique based on high-performance liquid chromatography-mass spectrometry (HPLC-MS) coupling, providing a theoretical basis for exercise to regulate the metabolism of Alzheimer’s disease (AD) organisms. Three-month-old male C57BL/6JNju mice, six wild-type (NC, *n* = 6); 12 APP/PS1 double transgenic species in total, were randomly divided into AD model (AM, *n* = 6) and AD model exercise (AE, *n* = 6) groups. The mice in the NC group were fed naturally, the mice in the AM group were statically placed on a running platform, and the mice in the AE group received a 20-week long-term moderate intensity running platform exercise intervention. Following the exercise intervention, the cecum contents of the mice in each group were collected and analyzed using the HPLC-MS technique, with those meeting both variable important in projection (VIP)> 1.5 and *p* < 0.05 being screened as differential metabolites. A total of 32 different metabolites were detected between the AM and NC groups, with 19 up-regulated in the AM group such as phosphatidic acid (PA) (18:4(6Z,9Z,12Z,15Z)/21:0) and 13 down-regulated in the AM group, such as 4,8-dimethylnonanoyl, compared to the NC group; 98 different metabolites were found between the AM and AE groups, 41 of which were upregulated such as Lyso phosphatidylcholine (LysoPC) and 57 of which were downregulated compared to the AM group such as Phosphatidylinositol (PI). The regulation of linoleic acid metabolism, glycerophospholipid metabolism, bile secretion, phenylalanine metabolism, and other pathways was predominantly regulated by nine metabolites, which were subsequently identified as indicators of exercise intervention to enhance metabolism in AD mice. The metabolomic technique can identify the metabolic problems of intestinal contents in AD mice and initially screen the biomarkers of exercise to improve the metabolic disorders in AD. These findings can help us better understand the impact of aerobic exercise on AD metabolism.

## 1. Introduction

There is one new instance of dementia detected every three seconds, according to a 2018 World Health Organization (WHO) report on AD [1], making it vital to lower incidence and lessen the pathophysiology of AD. Exercise may be a useful non-pharmacological strategy to prevent and treat AD, according to a wealth of epidemiological and experimental research. Previous studies in humans have shown that exercise is effective in lowering the risk of asymptomatic or preclinical AD and improving cognitive function in patients with mild AD. Huang summarizes that exercise improves cognitive function in normal aging and mild cognitive impairment (MCI) while making functional and structural changes in brain regions and suggests that the default mode network, the frontoparietal network, and the frontal executive network may be three of the most valuable targets for assessment of intervention efficiency [2]. Norton S uses relative risk estimates from existing meta-analyses to suggest that reducing the incidence of vascular risk through effective methods such as physical activity could reduce the incidence of Alzheimer’s disease [3]. Borges Machado F’s study shows that amulticomponent intervention that includes muscle strengthening, aerobic exercise, balance and postural exercises has a positive impact on patients with mild to moderate AD [4]. Arenaza-Urquijo’s study shows that physical activity in late adulthood is associated with gray matter volume in normal older adults, especially in aging and AD-susceptible areas [5].

Similarly, studies in animals have shown that exercise is effective in improving learning memory capacity in early AD brain [6,7,8,9] and reducing amyloid β-protein (Aβ) lesions [10,11]. However, the mechanism by which exercise improves AD learning memory is unknown.

The pathogenesis of AD may be influenced by non-Aβ processes like calcium dysregulation, mitochondrial dysfunction, altered cell signaling, oxidative stress, inflammation, and lipid homeostasis, according to mechanistic studies [12,13,14]. Recent research indicates that deficiencies in the gastrointestinal neural system may constitute the pathophysiology of neurodegenerative illnesses, including AD [15]. Trillions of bacteria make up the human intestinal ecology, which functions as a bioreactor powered by the abundance of nutrients in the food that produce bioactive molecules. These microbiota-derived compounds communicate with distant bodily organs, allowing gut bacteria interaction with the host’s immunological, hormonal, brain, and other systems [16]. The maintenance of a healthy host’s key functions depends on this microbial–host connection. Although exercise affects the microbiota in both people and animals [17,18,19], it is unknown whether these metabolic alterations in gut bacteria are related to the development of AD.

In order to identify possible biomarkers, APP/PS1 double transgenic model mice were chosen and their intestinal contents were collected for metabolomic analysis using the HPLC-MS method following exercise intervention.

## 2. Materials and Methods

### 2.1. Experimental Animals

**Figure 1:**
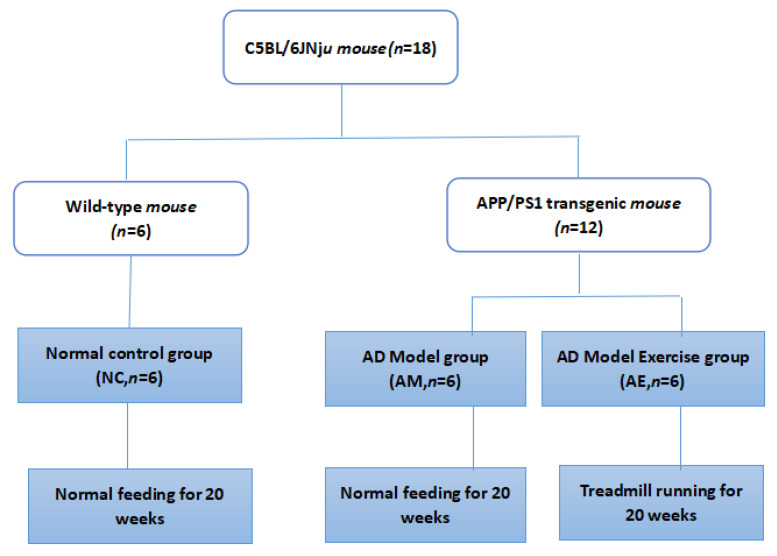
Experimental grouping and intervention protocol.

In separate cages of three mice each, 18 male C57BL/6JNju mice aged 3 months were housed, including 6 wild-type mice and 12 transgenic APP/PS1 animals (17–25 g). After one week of acclimatization, wild-type mice were used as the normal control group (NC group, *n* = 6). APP/PS1 double transgenic mice were randomly divided into two groups: an AD model group (AM group, *n* = 6) and an AD model exercise group (AE group, *n* = 6) (Figure 1). Mice were purchased from Nanjing University-Nanjing Institute of Biomedical Research (Nanjing, China). The experimental protocol was approved by the Institutional Animal Care and Use Committee of the Chengdu Sports Institute, approval number [2021]21, and the experimental groups and intervention protocols are depicted in Figure 1. Animal experiments were carried out in accordance with the National Research Council Guide for the Care and Use of Laboratory Animals (8th edition, 2011) [20]. Animal feeding conditions were as follows: clean and quiet environment, room temperature of 20–28 °C, relative humidity of 40–60%, simulated natural lighting conditions, 12 h of light per day, free intake of animal feed and pure water (pure water is provided by the water purification system in the animal experimental room), and weekly change of bedding, food and drinking water that were sterilized by ultraviolet rays.

### 2.2. Movement Scheme

Table 1 displays the exercise intervention protocol. The running platform (DSPT-208, Shanghai, China) was introduced to mice in the AE group over the course of two days for 10 min each, at speeds of 5 and 8 m per minute, respectively. After the adaptation, a formal exercise intervention lasting 20 weeks, five days per week, and 30 min per day was carried out. This involved exercising at speeds of 10 m/min for five minutes, 15 m/min for 20 min, and 10 m/min for the remaining five minutes. When the mice reached this level of exertion, there was no electrical stimulation and only a gentle touch on the tail was utilized to restart the exercise (Table 1) [21].

### 2.3. Morris Water Maze Behavioral Test

On the last week of the exercise intervention, the Morris water maze test (Huaibei Zhenghua, Anhui, China) was to evaluate the spatial learning and memory ability of mice; it is a classic method of studying rodent neuroscience [22]. The average latency of escape and the number of times of crossing the platform were used as the important indicators of spatial learning and memory in mice [23]. At the end of the last day of exercise intervention, the Morris Constant Temperature Water Maze System was used for behavioral training and testing. The training and test consisted of two parts, namely the “orientation navigation experiment” and the “space exploration experiment”. Positioning navigation experiments were carried out on a platform placed in the fourth quadrant of a circular pool. Mice were placed in the pool, facing any point on the wall of the pool. The video system automatically records the mouse’s swimming path and calculates exactly how long it takes each mouse to find the platform. After the mice found the platform, or if the mice failed to find the platform within 2 min, the mice were directed to the platform and allowed rest for 10–20 s before the next experiment; then, the next experiment was started. The mice were trained four times a day for six days, all in the dark. Then, the space exploration experiment was carried out 24 h after the end of the navigation test. the platform was removed and the mice were placed in the second quadrant of the pool. The time it took the mice to swim across the quadrant, where the platform used to be, was recorded.

### 2.4. Sample Collection and Processing

#### 2.4.1. Sample Collection

All mice were fasted for 12 h following the exercise intervention, and the experimental mice were given 0.5% sodium pentobarbital (50 mg/kg) to put them to sleep. The cecum’s contents were then rapidly removed and kept in a −80 °C freezer for metabolomic analysis.

#### 2.4.2. Pretreatment of HPLC-MS Samples

By aseptic manipulation, 25 mg of precisely weighed cecum contents was transferred into a 1.5 mL Ep tube. The tubes were filled with two tiny steel balls. Each sample received 20 μL of the internal standard (2-chloro-l-phenylalanine in methanol, 0.3 mg/mL), as well as 600 μL of the extraction solvent (4/1, *v*/*v*) methanol. The samples were stored at −20 °C for 2 min, then ground at 60 HZ for 2 min, sonicated at 0 °C for 10 min, and stored at −20 °C for 30 min. A crystal syringe was used to collect the supernatant (150 μL) from each tube, which was then transferred to LC vials after passing through a 0.22 μm microfilter. Prior to HPLC-MS analysis, the vials were kept at −80 °C. Aliquots from each sample were combined to create a pooled sample for QC samples.

#### 2.4.3. Chromatographic Conditions

For the analysis of positive and negative ions, a 1.7 μm, 100 × 2.1 mm ACQUITY UPLC BEH C18 column (Thermo Fisher Scientific, Waltham, MA, USA) was employed. The acetonitrile/methanol (2/3, *v*/*v*) and water (with 0.1% formic acid, *v*/*v*) used in the binary gradient elution system had the following gradients for separation: 5–20% B in 0–2 min, 20–25% B in 2–4 min, 25–60% B in 4–9 min, and 60–100% B in 9–17 min. Components were maintained at 100% B for 2 min, followed by 100% to 5% B at a flow rate of 0.4 mL/min and a column temperature of 45 °C for 19.1 to 20.1 min. Throughout the analysis, a temperature of 4 °C was maintained for all samples. The injection had a volume of 5 μ L. The resolution was 70,000 and the mass range was *m*/*z* 70 to 1000.

#### 2.4.4. Mass Spectrometry Conditions

The Waters ACQUITY UPLC system with an AB Triple TOF 5600 mass spectrometer (Thermo Fisher Scientific, Waltham, MA, USA) with a heated electrospray ionization (ESI) source for the analysis of metabolic signatures in both ESI-positive and ESI-negative ion modes. The HCD MS/MS (Thermo Fisher Scientific, Waltham, MA, USA) can resolution was 35,000, and the entire mass spectrometry scan resolution was 70,000. The 10 eV collision energy was chosen.

### 2.5. Data Analysis

Using Proqenesis QI V2.3 software (Nonlinear, Dynamics, Newcastle, UK), the raw HPLC-MS data were examined. The product tolerance was 10 ppm, the product ion threshold was 5%, and the precursor tolerance was 5 ppm. The three-dimensional data set in the Excel file that was obtained includes peak identification, peak matching, and peak alignment. Native QI (Waters Corporation, Milford, CT, USA) data processing software used public databases including HMDB, (http://www.hmdb.ca, accessed on 18 December 2018) and METLIN (http://www.lipidmaps.org/METLIN, accessed on 1 December 2017) to identify the metabolites. The normalized data were imported into R languages for (orthogonal) partial least squares discriminant analysis (O)PLS-DA to intuitively reflect the metabolic changes between the experimental groups. According to the statistical significance threshold value (Variable Important in Projections, VIP) of the effect of variables on projection generated from the OPLS-DA model, the substances with VIP > 1.5 were screened, and one-way ANOVA (SPSS 22.0, Amenk, NY, USA) was then conducted following the t-test. They were used as candidate biomarkers when both VIP > 1.5 and *p* value < 0.05 were satisfied.

Using the randomized forest approach, the NC and AM groups were analyzed for feature importance ranking, with Mean Decrease Accuracy as the assessment indicator.

## 3. Results

### 3.1. General Health Status of Animals

Mouse in NC and AE groups were in good mental state, had a normal diet, were agile, had shiny hair and healthy body. In the AM group, mouse showed fatigue, absence of appetite, low excretion, drowsiness, and obvious cognitive impairment symptoms, such as slow movement, hair loss, etc. (Table 2).

### 3.2. Behavioral Test—Morris Water Maze Experiment

In the Morris water maze navigation test, the results of the localization navigation experiment (Table 3, Figure 6a) showed that the mean escape latency of NC and AE mice gradually decreased with the increase in the number of training days, and the spatial memory of the platform position was initially formed on Day 3, but no stable spatial memory was formed in the AM group (*p* < 0.05 versus all other groups). The times of crossing platforms (Table 4, Figure 6b) was highest in Group C, followed by those in Group E. Group C values were significantly higher than those of Group M (*p* < 0.01). Group E values were significantly higher than those of Group M (*p* < 0.01).

### 3.3. Results of (O)PLS-DA Analysis

**Figure 2:**
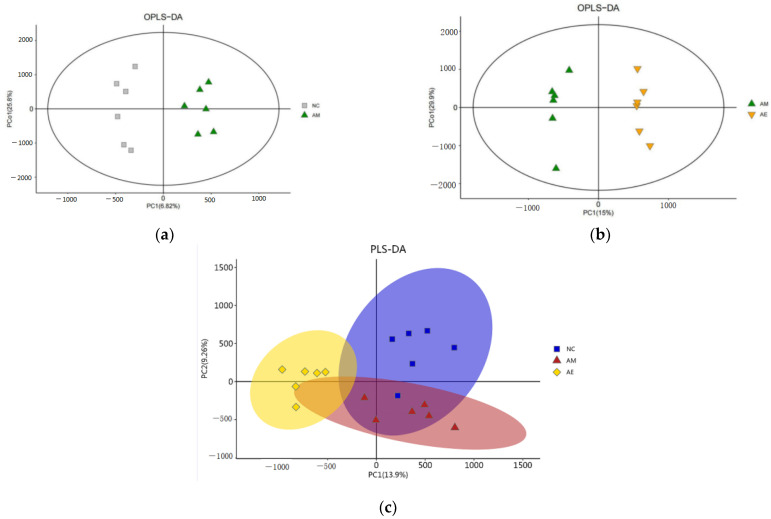
Different group (O)PLS-DA score chart. (**a**) NC/AM group OPLS-DA score chart. Note: Grey squares indicate NC group, green triangles indicate AM group. (**b**) AM/AE group OPLS-DA score chart. Note: Green triangles indicate the AM group, yellow triangles indicate the AE group. (**c**) NC/AM/AE group PLS-DA score chart. Note: Blue squares indicate NC group, red triangles indicate AM group, purple triangles indicate AE group.

The (O)PLS-DA score plots (Figure 2) revealed that samples were more concentrated within each group of mice, indicating good sample aggregation; mouse samples were significantly different between comparison groups; and the metabolites of mouse intestinal contents varied between groups. The generated differential metabolites can therefore reflect the biological variations between samples, and the test technique is trustworthy and stable.

### 3.4. Differential Metabolite Identification

**Figure 3:**
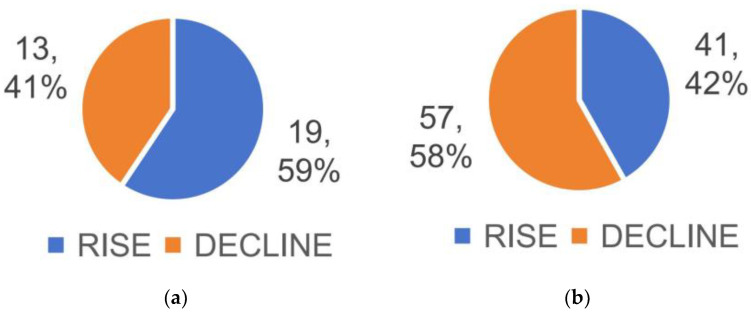
Number of differential metabolites in each group of mice. (**a**) Number of differential metabolites in AM/NC group. (**b**) Number of differential metabolites in AE/AM group.

**Figure 4:**
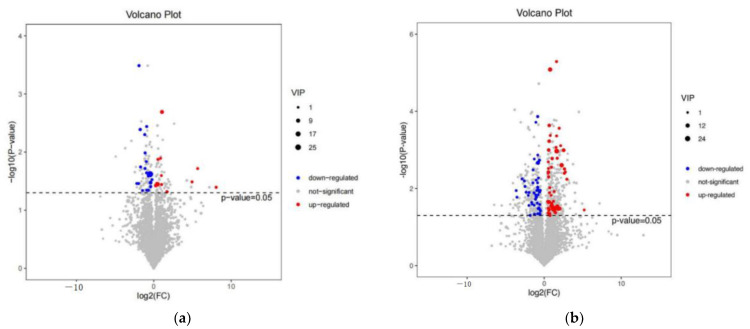
Volcano plot of differential metabolites in different groups. (**a**) Volcano plot of differential metabolites in NC/AM group. Note: Red indicates up-regulated differential metabolites, blue indicates down-regulated differential metabolites, and gray indicates no differential metabolites. (**b**) Volcano plot of differential metabolites in AM/AE group. Note: Red indicates up-regulated differential metabolites, blue indicates down-regulated differential metabolites, and gray indicates no differential metabolites.

By screening for metabolic differentials between the two groups that satisfied both VIP > 1.5 and *p*-value < 0.05, 32 differential metabolites were detected between the AM and NC groups, with 19 up-regulated and 13 down-regulated in the AM group compared to the NC group (Figure 3a and Figure 4a); between the AM and AE groups, a total of 98 differential metabolites were detected, with 41 up-regulated and 57 down-regulated (Figure 3b and Figure 4b, Table S1).

Finally, nine possible biomarkers were obtained based on differential metabolite screening (Table 5).

### 3.5. Results of Mean Decrease Accuracy

**Figure 5:**
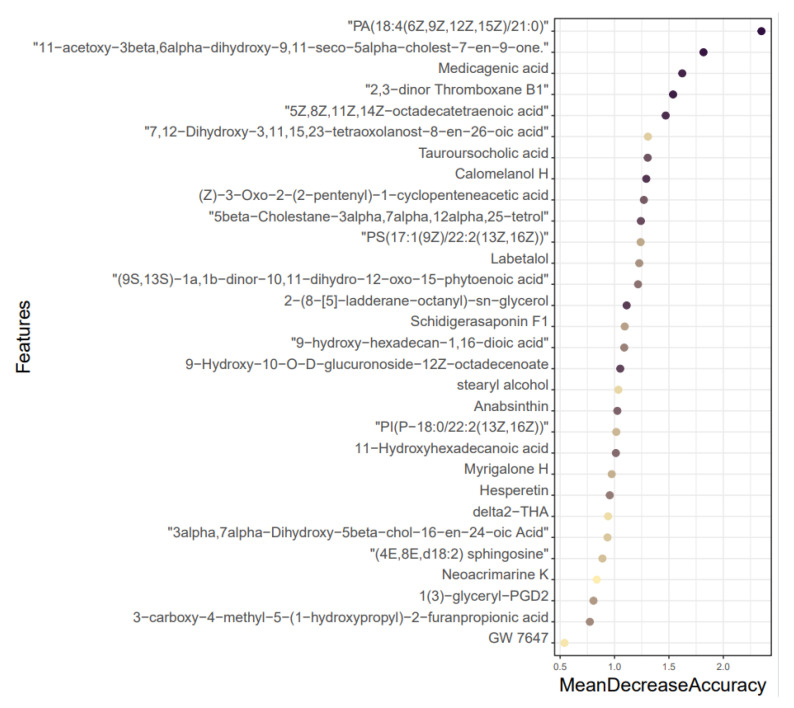
Mean Decrease Accuracy sorting chart.

Using a random forest approach with MeanReducedAccuracy as the assessment metric, the 30 metabolites with the highest priority between the NC and AM groups had the highest characteristic importance of PA (18:4(6Z,9Z,12Z,15Z)/21:0) (Figure 5).

## 4. Discussion

This study is the first to examine the impact of exercise on intestinal contents in AD mice using an HPLC-MS method. We analyzed the metabolome of intestinal contents in wild-type mice, AD mice, and AD mice after exercise intervention, and then we screened nine differential metabolites (Table 5). These metabolites primarily involve lipid metabolism, bile secretion, phenylalanine metabolism, and other pathways.

### 4.1. Exercise Can Improve the Ability of Learning and Memory in AD Mice

**Figure 6:**
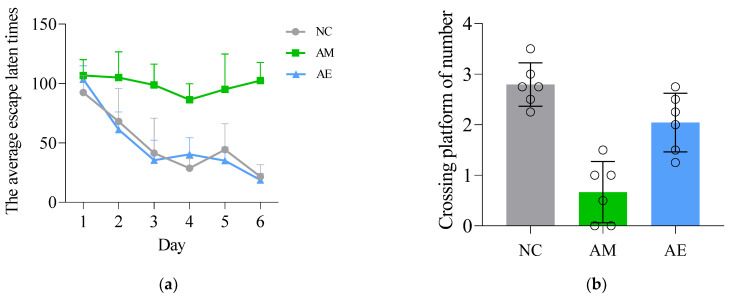
Results of the Morris water maze experiment. (**a**) Evaluation of navigation ability of mice: Mean escape latency time of mice in each group. (**b**) Assessment of the spatial exploration ability of mice, the number of times of crossing the platform. Data are expressed as mean ± standard deviation (M ± SD) *(n* = 6).

AD is the most common senile neurodegenerative disease and the main type of dementia. Its physiological characteristics include cognitive impairment, neurodegeneration, amyloid beta protein (Aβ) deposition, neurofibrillary tangles formation, and neuroinflammation. In action, it shows changes in learning and memory functions [24]. Physical exercise has a protective effect on cognitive function in older adults with neurodegenerative diseases, including Alzheimer’s disease [25]. Our results showed that, with the increase in training times, the average escape latency of each group gradually decreased. On the third day of the Morris water maze test, the escape latency of NC and AE groups was significantly shortened (Figure 6a), indicating that the NC and AE groups formed stable spatial memory on the third day, while the AM group did not. Compared with the NC group, the space exploration ability (the number of crossing platforms) of the AM group was poor, while the AE group had no difference (Figure 6b). Our behavioral experiment results showed that compared with the NC group, the learning and memory ability of the AM group was significantly decreased, while 20 weeks of aerobic exercise could significantly improve the spatial learning and memory ability of AD model mice.

### 4.2. Effect of Exercise on Lipid Metabolism in AD Mice

By comparing the metabolic profiles of AM with the AE group, it was found that exercise had the greatest effect on the metabolism of lipids and lipid-like molecules in AD mice. 4,8 dimethylnonanoyl carnitine is an intermediate generated by a series of reactions between phytanic acid and calcitonin after being oxidized in the peroxisome and eventually entering the mitochondria, playing an important role in fatty acid β-oxidation. Zhang Z et al. showed that 4,8 dimethylnonanoyl carnitine played an important role in the AD model. The expression of 4,8 dimethylnonanoyl carnitine was decreased in serum of rats [26], similar to their findings, and the present study found that AD decreased its expression, while exercise increased it, suggesting that exercise contributes to the improvement of fatty acid β-oxidation in AD. Antioxidant enzymes such as superoxide dismutase and catalase are decreased in the frontal and temporal cortex of brain tissue of AD patients [27], while exercise can regulate the metabolic levels of AD peroxidase [28], suggesting that exercise may affect fatty acid β-oxidation by improving AD peroxisome activity and thereby.

Glycerophospholipids are polar lipids that are ubiquitous in all tissues and are essential components of cell membranes [29]. PA regulates various steps of synaptic vesicle transport in neurons and plays an important role in neurotransmission [30], and is also important for Aβ production [31]; PS mediates synaptic autophagy pathways and is involved in Aβ signaling in AD [32]; Wollen et al. demonstrated that PS protects neurons by inhibiting Aβ and inflammation [33]; PE is enriched in mitochondrial membranes and has the function of regulating autophagy [34], and some studies have shown that serum levels of PE can predict the time to progression of mild cognitive dysfunction to AD in humans [30]. Numerous studies have demonstrated abnormal phospholipid metabolism in AD mice [35], and similar to their findings, the present study showed that PA(18:4(6Z,9Z,12Z,15Z)/21:0) expression was increased, and PS(17:1(9Z)/22:2(13Z,16Z), PE(14:0/22:2(13Z,16Z)) expressions were decreased in AD mice, indicating that the disorders of glycerophospholipid metabolism may be one of the important pathological manifestations of AD, and after exercise, PA(18:4(6Z,9Z,12Z,15Z)/21:0) expression was decreased (*p* < 0.01), and PS(17:1(9Z)/22:2(13Z,16Z) (*p* < 0.01), PE(14:0/22:2(13Z,16Z)) expressions were elevated (*p* < 0.01), suggesting that mice’s glycerophospholipid metabolism was altered by long-term moderate intensity aerobic exercise. The metabolism of glycerophospholipids is mainly catalyzed by phospholipases, of which phospholipase A2 is considered to be a key marker for the development of AD [36], and exercise-induced abnormalities in glycerophospholipid metabolism in AD mice may be related to changes in their activity [37,38]. In the interaction between -6 linoleic acid and reactive oxygen species, 13-hydroxyoctadecadienoic acid (13-HODE) is a mediator that has the potential to inhibit cell proliferation, trigger cell apoptosis, cause endoplasmic reticulum stress, induce oxidative stress, and disturb lipid homeostasis [39,40]. In the current study, we discovered that 13-HODE expression was increased in AD mice and it lowered with exercise, indicating that exercise enhances AD linoleic acid oxidation. Notably, utilizing an acute pro-inflammatory exercise model, David C. Nieman et al. discovered a substantial elevation of 13-HODE expression in mice [41], indicating that 13-HODE is positively linked with the inflammatory response. This discrepancy might be explained by the type of exercise, as chronic aerobic exercise reduces inflammation whereas acute high-intensity exercise actually promotes acute inflammation. As a result, we speculate that regular aerobic exercise may be helpful in lowering the inflammatory reaction linked to AD linoleic acid oxidation.

### 4.3. Effect of Exercise on Bile Metabolism of Intestinal Contents in AD Mice

The primary method by which the liver eliminates cholesterol is by cholesterol production in hepatocytes. Bile acids (BA) are created in the liver from cholesterol. Primary and secondary bile acids are the two forms of BA. As primary bile acids enter the colon, they are catalyzed by intestinal bacterial enzymes to transform into secondary bile acids by reactions such defixation, dehydrogenation, dehydroxylation, and sulfate esterification. Disproportionate ratios of primary and secondary BA in serum and brain samples from AD mice have been documented in the literature to be caused by the complicated interactions between BA, gut microbiota, and host metabolism [42]. Therefore, there is growing interest in the dysfunction of the brain–gut–microbiota axis in the pathophysiology of AD. In the current work, the higher expression of 5β-Cyprinolsulfate, a primary bile acid biosynthesis intermediate, in the AM group raises the possibility that AD mice have defective primary bile acid metabolism, which is improved by exercise. We hypothesize that bile acid metabolism may be a target of exercise-induced gut microbial metabolism to enhance AD in light of a previous study that showed a reduction in total plasma bile acids after a 1 h resistance exercise in 10 healthy adults [43].

The primary pigment of human bile, bilirubin, is produced from heme in red blood cells, carried by the bloodstream to the liver, and then entering the intestine where it is broken down by intestinal microbes into urobilinogen and expelled in bile and feces. Unconjugated bilirubin is thought to be a potentially significant antioxidant and neuroprotective agent since sustained high levels of unconjugated bilirubin are linked to neurotoxicity, but moderate levels of unconjugated bilirubin have a preventative effect on several disorders [44,45], and therefore bilirubin has been recognized as a potentially important antioxidant and neuroprotective agent [46]. The current study finds that exercise raises bilirubin and urobilin levels in AD mice, whereas bilirubin and urobilin levels are decreased in AD animals, suggesting that bilirubin metabolism is disrupted in AD mice. It is hypothesized that exercise helps AD mice’s antioxidant levels.

### 4.4. Effect of Exercise on Phenylalanine Metabolism in AD Mice

Numerous studies have demonstrated that phenylalanine metabolism is dysregulated in AD patients, and Liu P. et al. [47] used 44 postmortem samples from AD patients in untargeted and targeted metabolomics experiments and discovered that dysregulated phenylalanine metabolism was connected to AD pathogenesis. The present study’s findings, which are consistent with those of a recent study by G Wan et al. [48], demonstrated that exercise changed the phenylalanine metabolism in AD mice. Phenylalanine was shown to be down-regulated in the intestinal contents of AD mice, whereas exercise boosted its expression. Phenylalanine hydroxylase catalyzes the majority of phenylalanine in the body to create tyrosine, which is essential for the production of hormones and neurotransmitters including dopamine and adrenaline. Exercise has been shown to partially prevent and improve this process. Dopamine levels are significantly lower in AD patients, despite the fact that dopamine is essential to the mechanism underlying synaptic plasticity [49]. Synaptic disorders and neurotransmission impairment can also result in extracellular A deposition, senile plaques, and intracellular fiber tangles, which cause pre-dementia symptoms like cognitive decline. Exercise therefore improves the pathophysiology of AD animals, and one possible mechanism for this is the control of dopamine metabolism by the microbial–gut–brain axis.

## 5. Conclusions

In this study, a metabolomic approach was used to identify intestinal content metabolism disorders in AD mice, and preliminary screening was carried out to identify long-term aerobic exercise biomarkers to improve AD metabolic disorders that are related to the pathways of linoleic acid metabolism, glycerophospholipid metabolism, bile secretion, and phenylalanine metabolism. Further research is required to determine the ways in which exercise enhances cognitive function by mediating the effects of these biomarkers on the AD neurological system via the gut–brain axis. In the future, metabolites such as PA (18:4(6Z,9Z,12Z,15Z)/21:0), 4,8-dimethylnonanoyl, and PS (17:1(9Z)/22:2(13Z,16Z)) can be studied in a focused manner. These findings can help us better understand the impact of aerobic exercise on AD metabolism.

## 6. Limitations of Study

The main limitation of this paper concerns the number of samples. Fortunately, the current sample size is adequate for the purpose of this stage of the study, providing evidence of effective metabolic markers for the prevention of learning and memory in Alzheimer’s disease by exercise.

## Figures and Tables

**Table 1:** Exercise intervention program.

Time	Day 1	Day 2	Formal Campaign Interventions (20 Week)		
Speed (m/min)	5	8	10	15	10
Time (min)	10	10	5	20	5

**Table 2:** Description of general health status of mice at the end of the experiment.

Group	Average Weight (Mean ± SD)	Hair	Appetite	Excreta
AE	21 ± 2.45	Normal	Normal	Normal
AM	20 ± 1.65	Sparse	Low	Low
NC	24 ± 2.3	Normal	Normal	Normal

**Table 3:** The effect of location navigation experiment on the mean escape latency of mice (mean ± sd).

	NC	AM	AD
Day1	92.44 ± 14.16	106.75 ± 13.39	103.64 ± 11.24
Day2	68.84 ± 27.75	104.92 ± 21.79	61.11 ± 14.92 ▲
Day3	41.38 ± 29.39	98.73 ± 17.57 ■	35.33 ± 16.88 ▲▲
Day4	28.60 ± 8.03	86.34 ± 13.49 ■■	40.29 ± 13.97 ▲▲
Day5	44.31 ± 21.86	95.04 ± 29.79 ■	35.04 ± 11.57 ▲▲
Day6	21.49 ± 10.13	102.39 ± 15.26 ■■	18.79 ± 5.58 ▲▲

Note: ■: *p* < 0.05, compared with NC group; ■■: *p* < 0.01 compared with NC group; ▲: *p* < 0.05 compared with AM group; ▲▲: *p* < 0.01 compared with AM group.

**Table 4:** The times of mice in each group crossing the platform (mean ± sd).

Groups	Times of Crossing the Platform
NC	2.96 ± 0.60
AM	0.67 ± 0.55 ■■
AE	2.01 ± 0.48 ▲

Note: *p* < 0.05, compared with NC group; ■■: *p* < 0.01, compared with NC group; ▲: *p* < 0.05 compared with AM group.

**Table 5:** Potential intestinal content metabolites for exercise to improve AD pathology.

No.	Metabolites	Amount of Expression			AM/NC		AE/AM	
		NC	AM	AE	VIP	*p*-Value	VIP	*p*-Value
1	PA(18:4(6Z,9Z,12Z,15Z)/21:0)	96.45	363.70	118.30	2.81	0.000	1.82	<0.001
2	4,8-dimethylnonanoyl	359.19	177.77	481.43	1.91	0.036	1.90	0.003
3	PS(17:1(9Z)/22:2(13Z,16Z))	318.16	173.47	365.02	1.63	0.013	1.59	<0.001
4	13(S)-HODE	2320.98	3202.33	1940.018	-	-	3.26	0.04
5	5beta-Cyprinolsulfate	4356.15	1570.77	2802.11	-	-	3.54	0.02
6	Bilirubin	4845.45	618.18	920.51	-	-	7.49	<0.001
7	D-Urobilinogen	1157.84	491.56	709.93	-	-	3.72	0.04
8	PE(14:0/22:2(13Z,16Z))	2616.74	953.76	1119.14	-	-	1.95	0.01
9	Phenylacetylglycine	1298.78	245.91	815.51	-	-	2.09	<0.001

Note: VIP: variable weight value, from the VIP value of the OPLS-DA model; the larger the VIP, the greater the contribution of the variable to the grouping; *p*-value: the result of the t-test used to evaluate whether the variables differ significantly between the two groups of samples, *p* < 0.05 indicates significant.

## Data Availability

The data presented in this study are available within the article. Patients’ information is restricted due to privacy and ethical considerations.

## Supplementary Materials

The following supporting information can be downloaded at: https://www.mdpi.com/article/10.3390/metabo13111150/s1, Table S1: Metabolic differentials in the AE/AM group.
