# Supplementary material for: Mass Spectrometry Chromatography-Based Metabolomics: The Effect of Long-Term Aerobic Exercise on Learning Ability and the Metabolism of Intestinal Contents in Mice with Alzheimer’s Disease
Source: Metabolites. 2023 Nov 14;13(11):1150. doi: 10.3390/metabo13111150 (PMC10673277; doi:10.3390/metabo13111150)
Supplement: Supplementary file 1 [file metabolites-13-01150-s001.zip › metabolites-2680473-supplementary.pdf]

Supplemental Information

Table S1. Metabolic differentials in the AE/AM group.

Categories	No.	Metabolites	AE/AM			
			log2(FC)	*m/z*	VIP	*p* value
Lipids and lipid-like molecules	1	LysoPC(22:5(7Z,10Z,13Z,16Z,19Z))	0.57	552.35	2.17	0.05
	2	PE(14:0/22:2(13Z,16Z))	0.89	761.58	2.23	0.02
	3	PE(18:3(9Z,12Z,15Z)/18:0)	0.83	742.53	1.6	<0.01
	4	PI(O-16:0/20:1(11Z))	−1.04	895.59	4.76	<0.01
	5	PC(20:1(9Z)/0:0)	0.86	572.37	1.75	0.01
	6	PA(P-16:0/17:2(9Z,12Z))	1.32	665.45	2.49	0.01
	7	PS(O-20:0/22:0)	1.46	844.68	2.47	0.01
	8	PG(O-20:0/14:0)	−1.21	781.56	2.42	0.02
	9	LysoPE(16:1(9Z)/0:0)	1.82	452.28	2.19	0.05
	10	PA(20:4(5Z,8Z,11Z,14Z)/15:0)	1.63	665.45	1.52	0.02
	11	PA(15:0/20:5(5Z,8Z,11Z,14Z,17Z))	2.49	681.45	2.06	0.01
	12	PA(18:4(6Z,9Z,12Z,15Z)/21:0)	−1.62	756.56	1.82	<0.01
	13	PE(P-18:1(9Z)/22:6(4Z,7Z,10Z,13Z,16Z,19Z))	3.53	791.57	1.81	0.02
	14	PE(P-18:0/22:6(4Z,7Z,10Z,12E,16Z,19Z)(14OH))	3.63	809.58	1.73	0.01
	15	1-(2-methoxy-7Z,21Z-octacosadienyl)-sn-glycero-3-phosphoethanolamine	2.34	656.46	1.71	0.01
	16	PG(16:0/18:0)	−1.81	795.54	1.66	0.04
	17	PS(17:1(9Z)/22:2(13Z,16Z))	1.07	810.56	1.59	<0.01
	18	PS(14:1(9Z)/16:1(9Z))	1.98	686.44	1.54	0.03
	19	PA(P-18:0/17:2(9Z,12Z))	2.06	709.46	1.52	0.02
	20	13(S)-HODE	−0.66	295.23	3.26	0.04
	21	cis-gondoic acid	−1.47	309.28	3.72	0.04
	22	xi-10-Hydroxyoctadecanoic acid	−0.97	299.26	14.26	0.03
	23	12-Hydroxy-12-octadecanoylcarnitine	−0.28	466.35	1.59	0.01
	24	16-hydroxy stearic acid	−0.98	283.26	4.55	0.03
	25	Dehydrofalcarinone	0.83	498.34	3.13	0.01
	26	6-[3]-ladderane-1-hexanol	−1.7	263.24	2.97	<0.01

27	11-hydroperoxy-12,13-epoxy-9-octadecenoic acid	1.25	346.26	2.9	0.05
28	2-(9R-(5Z,9Z-tetracosadienoyloxy)-3-methyl-2Z-decenoyloxy)-ethanesulfonic acid	2.57	672.46	2.59	0.01
29	13-Heptadecyn-1-ol	−1.95	253.25	2.57	<0.01
30	Annohexocin	1.96	651.44	2.24	0.01
31	Tetranor-5-NO2-CLA	2.87	252.16	2.2	0.01
32	4,8 dimethylnonanoyl carnitine	1.44	330.26	1.9	<0.01
33	DG(18:3n6/0:0/20:4n6)	1.28	656.52	1.56	<0.01
34	Citronellyl trans-2-methyl-2-butenoate	0.78	219.18	1.55	0.01
35	5beta-Cyprinolsulfate	−0.56	513.29	3.54	0.02
36	5beta-scymnol sulfate	−0.64	547.29	1.96	0.04
37	Proscillaridin A	−0.66	548.32	5.84	<0.01
38	Tauro-b-muricholic acid	−0.61	514.28	4.83	0.02
39	(6RS,24R)-24,25-dihydroxyvitamin D3 6,19-sulfur dioxide adduct / (6RS,24R)-24,25-dihydroxycholecalciferol 6,19-sulfur dioxide adduct	0.55	498.32	2	0.05
40	7-Sulfocholic acid	−1.26	487.24	34.63	0.03
41	Cortolone-3-glucuronide	−2.26	581.23	12.93	<0.01
42	Homodolicholide	−0.61	473.32	1.61	0.05
43	Chenodeoxycholic acid sulfate	−1.7	471.24	9.03	0.03
44	Physapubenolide	−0.56	546.31	6.36	<0.01
45	23S,25,26-Trihydroxyvitamin D3	−0.58	450.36	5.89	<0.01
46	Physapubescin	−0.59	548.32	3.9	0.01
47	7alpha-Hydroxy-3-oxo-5beta-cholan-24-oic acid	−0.54	435.27	3.11	0.03
48	Physagulin F	−0.71	543.26	3	0.02
49	Corchoroside B	−0.94	499.27	2.76	<0.01
50	dolichyl diphosphate	−0.79	501.25	2.09	<0.01
51	Phorbol	−0.7	409.19	2.38	0.01
52	7,12-Dihydroxy-3,11,15,23-tetraoxolanost-8-en-26-oic acid	−0.77	529.28	11.92	<0.01
53	Ganoderic acid theta	−0.66	529.28	6.14	<0.01
54	Ganoderic acid N	−0.55	511.27	1.96	0.02
55	(-)-Euphomine A	−0.51	532.33	2.65	0.04
56	Pisumoside B	−1.3	675.32	2.09	0.01

	57	N-3,7-Dimethyl-2,6-octadienylcyclopropylcarboxamide	0.57	481.32	1.88	0.03
	58	4,5-(methanoxyethano)isolongifol-4-ene	0.84	278.25	1.77	0.05
	59	DG(18:3(9Z,12Z,15Z)/24:0/0:0)	0.84	741.58	3.11	0.00
	60	2-(8-[5]-ladderane-octanyl)-sn-glycerol	−1.54	385.27	2.05	0.00
	61	MGDG(18:3(9Z,12Z,15Z)/16:3(7Z,10Z,13Z))	0.97	764.53	1.73	0.01
	62	Cerasinone	−1.40	329.10	2.80	0.04
	63	Neolinderatone	0.82	511.32	2.21	0.01
	64	Kuhlmanniquinol	−0.43	334.16	1.51	0.00
	65	4-Hydroxy-5,7,4'-trimethoxyflavan	−0.43	315.12	1.51	0.01
	66	PI-Cer(d20:0/16:0)	0.61	810.58	3.31	0.00
	67	PI-Cer(t18:0/18:0(2OH))	0.80	824.57	1.76	0.03
	68	MG(0:0/20:5(5Z,8Z,11Z,14Z,17Z)/0:0)	−0.43	421.26	1.74	0.02
Benzenoids	69	Phenylacetylglycine	−0.72	385.14	1.98	0.05
	70	N-Ethylacetamide	0.6	88.08	2.14	0
	71	Aspartyl-Tryptophan	−2.62	656.27	1.85	0
Homogeneous non-metal compounds	72	Peroxynitrite	0.8	61.99	10.56	0
Hydrocarbons	73	7-Ethyl-3,6-dihydro-1,4-dimethylazulene	0.54	411.24	2.41	0.04
Lignans, neolignans and related compounds	74	Enterolactone	−0.62	299.13	2.44	0.04
Organic acids and derivatives	75	Phenylacetylglycine	−0.72	385.14	2.28	0.01
	76	N-Ethylacetamide	0.6	88.08	4.12	0.01
	77	Aspartyl-Tryptophan	−2.62	656.27	2.98	0
Organic compounds	78	Bilirubin	−2.53	583.25	7.49	0
Organic nitrogen compounds	79	Diethanolamine	0.75	88.08	1.95	0.01
	80	Phytosphingosine	−1.6	318.3	2.6	0
Organic oxygen compounds	81	(±)-(Z)-2-(5-Tetradecenyl)cyclobutanone	−0.98	265.25	1.67	0.02
Organoheterocyclic compounds	82	D-Urobilinogen	1.04	589.3	2	0.01
	83	Pentoxifylline	−2.14	557.28	1.73	0
	84	Alosetron	−0.54	589.3	2.04	0
	85	5,8-dihydroxy-2-(1-hydroxy-3-methoxy-4-oxocyclohexyl)-3,7-dimethoxy-4H-chromen-4-one	−0.88	425.11	1.97	0.03

	86	2-Oxindole-3-acetate	−1	381.11	1.5	0.03
Phenylpropanoids and polyketides	87	Licoricidin	−1.82	469.22	1.65	0.03
	88	(4-{2-[(1R,16Z,24E,26E,28Z)-1,18-dihydroxy-19,30-dimethoxy-15,17,21,23,29,35-hexamethyl-2,3,10,14,20-pentaoxo-11,36-dioxa-4-azatricyclo[30.3.1.$0^{4,9}$]hexatriaconta-16,24,26,28-tetraen-12-yl]propyl}-2-methoxycyclohexyl)oxidanesulfonic acid	−1.99	992.51	9.27	0.03
	89	(2Z)-2-[(3hydroxyphenyl)methylidene] octanoic acid	−1.47	495.28	1.58	0.03
Alkaloids and derivatives	90	Homoarecoline	1.13	339.23	1.95	0.02
Unclassified	91	cis-9,10-Epoxystearic acid	−1.63	299.26	16.9	0
	92	5-NITRO-2-PHENYLPROPYLAMINOBENZOIC ACID [NPPB]	−2.63	283.11	4.36	0
	93	3-hexanoyl-NBD Cholesterol	1.02	663.45	3.91	0.02
	94	3α,6α-Dihydroxy-7-oxo-5β-cholan-24-oic Acid	−0.75	407.28	3.35	0.05
	95	Polyporusterone A	0.61	479.34	3.29	0.05
	96	N-Acetyl-leu-leu-leu-leu-leu-tyr-amide	−2.94	788.53	2.6	0.01
	97	ApNA	0.47	425.28	2.22	0.04
	98	Evobioside	−5.2	683.36	1.82	0.04

Note: VIP: variable weight value, from the VIP value of the OPLS-DA model, the larger the VIP, the greater the contribution of the variable to the grouping; *p* value: the result of the t-test, used to evaluate whether the variables differ significantly between the two groups of samples, $p<0.05$ indicates significant; Log2(FC): the ratio of the mean expression of metabolites in the two groups of samples, a positive value indicates upregulation, a negative value indicates down-regulation.
